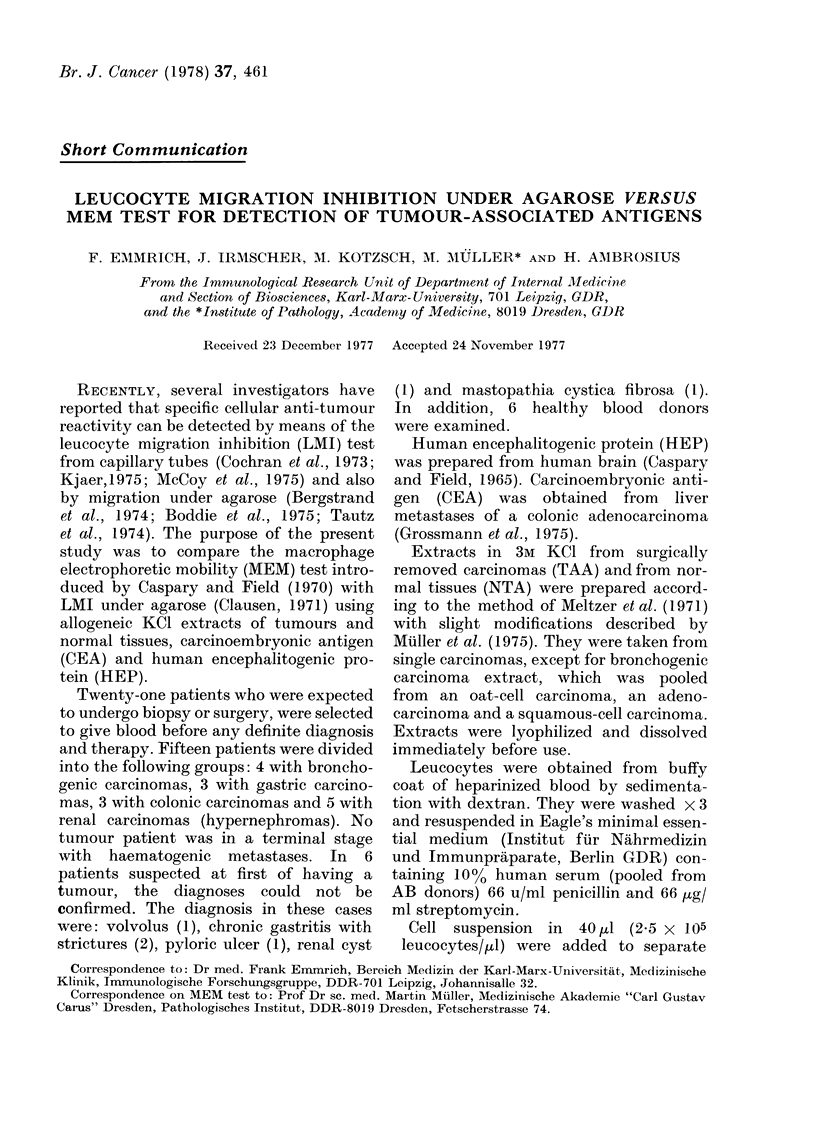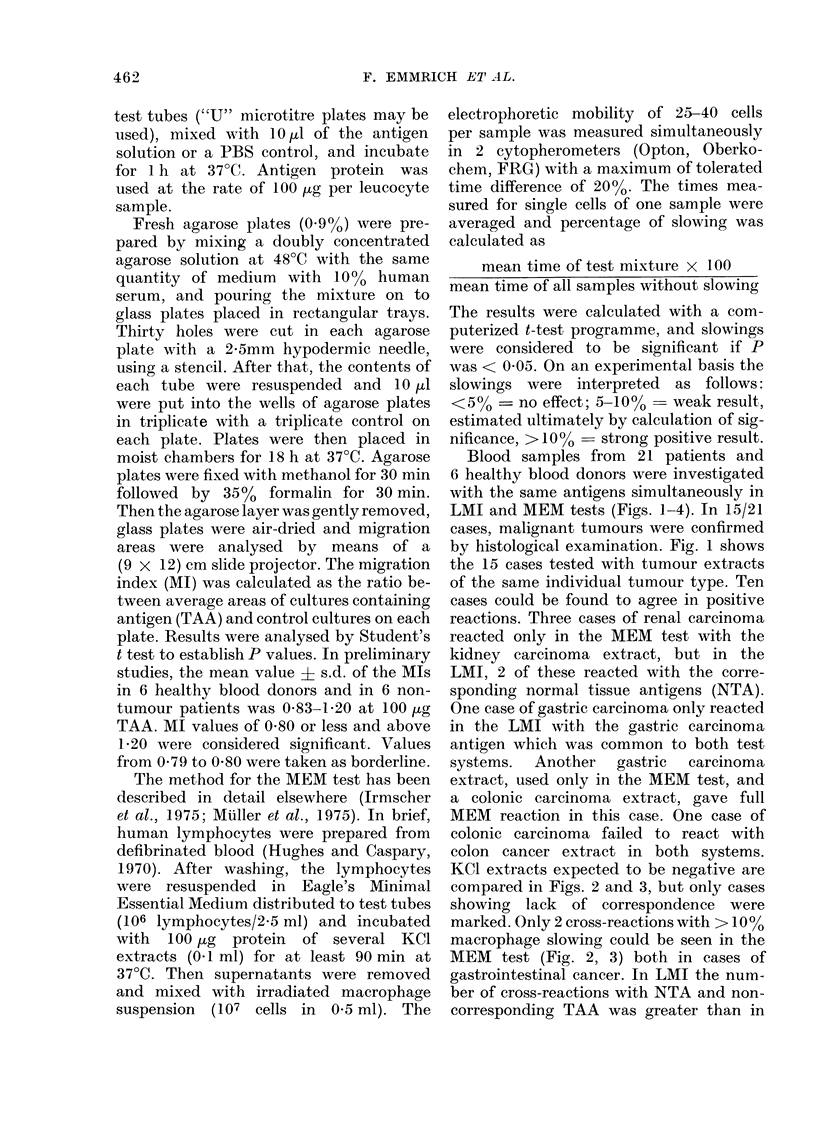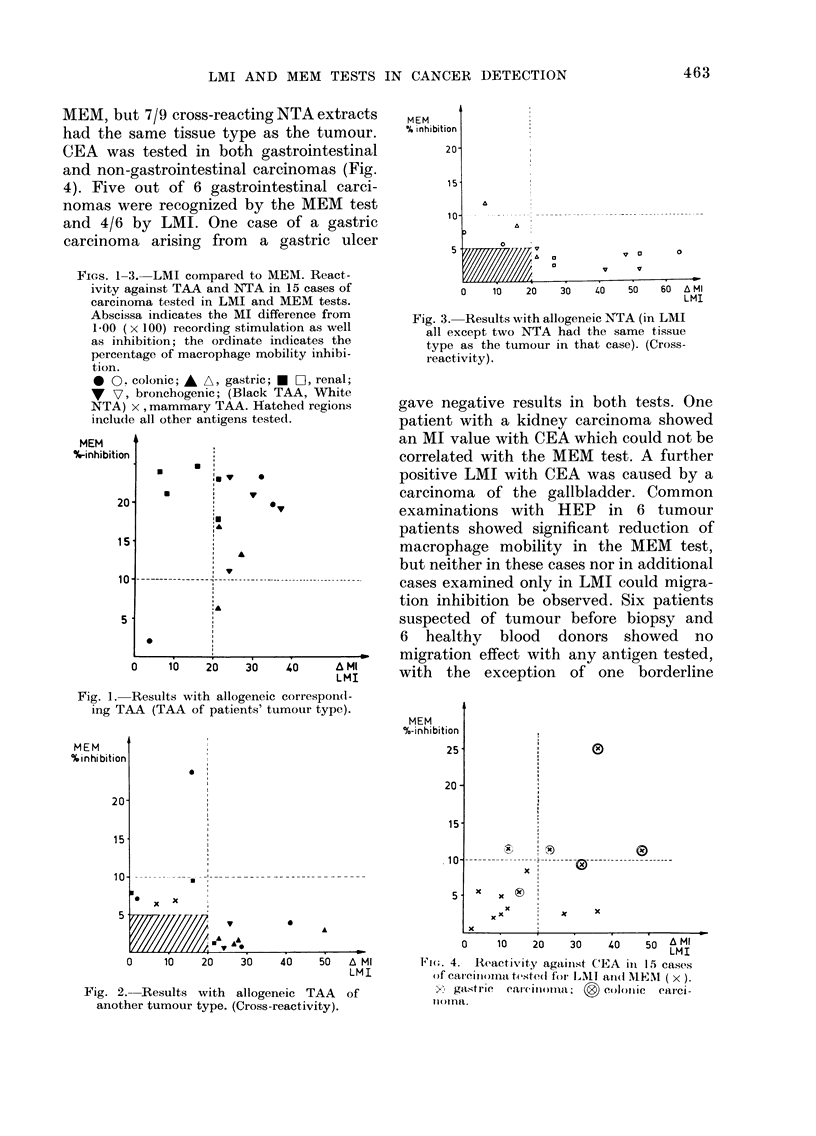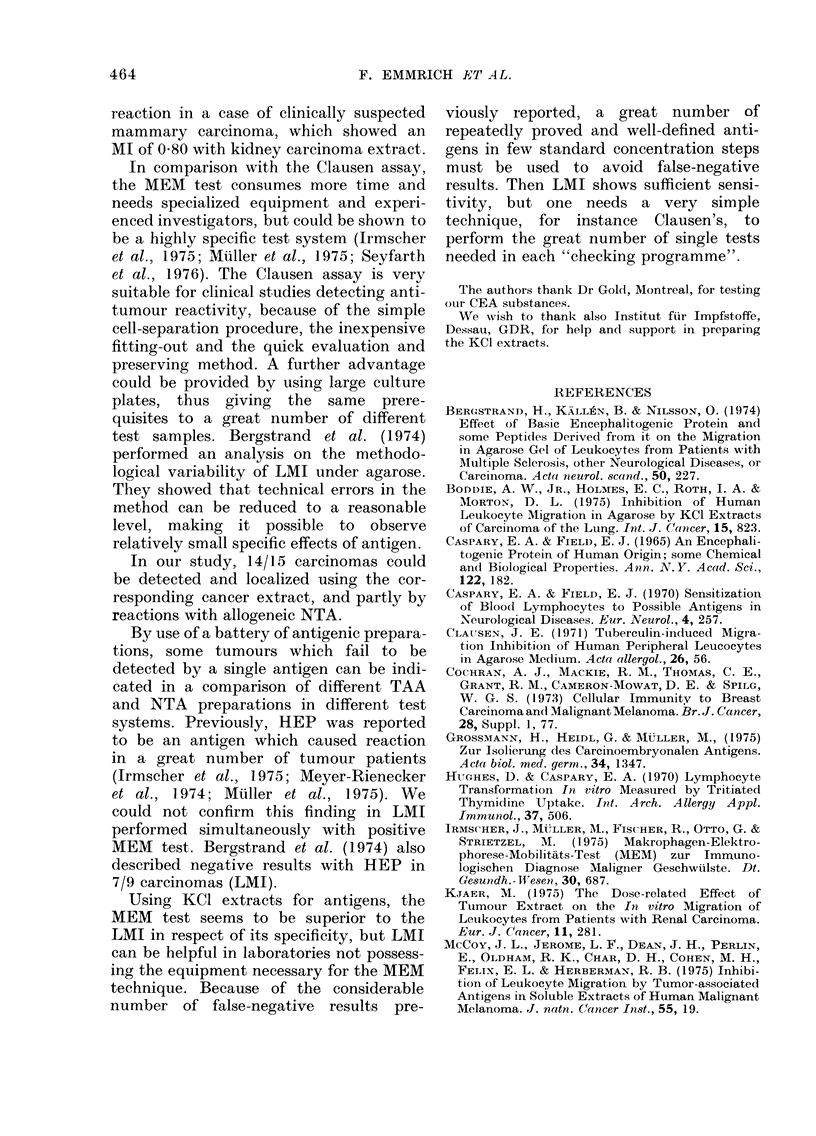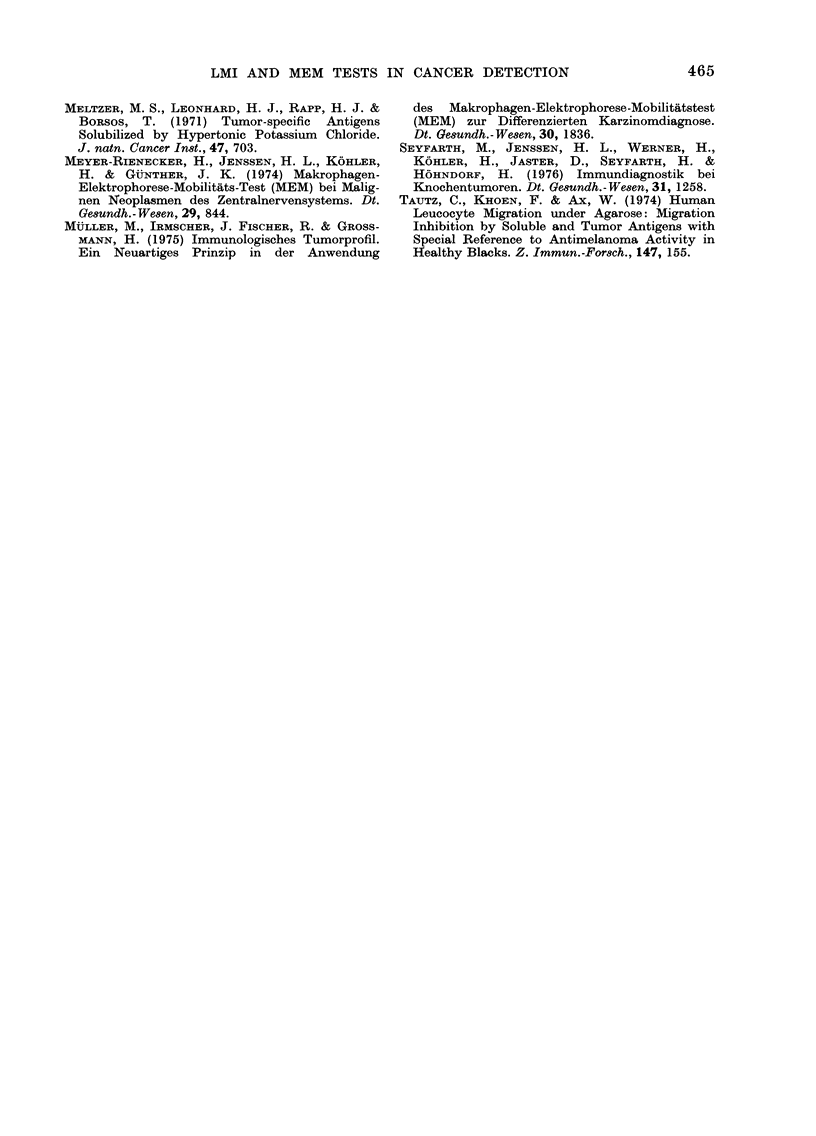# Leucocyte migration inhibition under agarose versus MEM test for detection of tumour-associated antigens.

**DOI:** 10.1038/bjc.1978.67

**Published:** 1978-03

**Authors:** F. Emmrich, J. Irmscher, M. Kotzsch, M. Müller, H. Ambrosius


*Br. J. Cancer* (1978) **37**, 461

***Short Communication***

### LEUCOCYTE MIGRATION INHIBITION UNDER AGAROSE *VERSUS* MEM TEST FOR DETECTION OF TUMOUR-ASSOCIATED ANTIGENS

F. EMMRICH, J. IRMSCHER, M. KOTZSCH, M. MÜLLER* AND H. AMBROSIUS

*From the Immunological Research Unit of Department of Internal Medicine and Section of Biosciences, Karl-Marx-University,* 701 *Leipzig, GDR, and the *Institute of Pathology, Academy of Medicine,* 8019 *Dresden, GDR*

Received 23 December 1977 Accepted 24 November 1977

RECENTLY, several investigators have reported that specific cellular anti-tumour reactivity can be detected by means of the leucocyte migration inhibition (LMI) test from capillary tubes (Cochran *et al.*, 1973; Kjaer,1975; McCoy *et al.*, 1975) and also by migration under agarose (Bergstrand *et al.*, 1974; Boddie *et al.*, 1975; Tautz *et al.*, 1974). The purpose of the present study was to compare the macrophage electrophoretic mobility (MEM) test introduced by Caspary and Field (1970) with LMI under agarose (Clausen, 1971) using allogeneic KCl extracts of tumours and normal tissues, carcinoembryonic antigen (CEA) and human encephalitogenic protein (HEP).

Twenty-one patients who were expected to undergo biopsy or surgery, were selected to give blood before any definite diagnosis and therapy. Fifteen patients were divided into the following groups: 4 with bronchogenic carcinomas, 3 with gastric carcinomas, 3 with colonic carcinomas and 5 with renal carcinomas (hypernephromas). No tumour patient was in a terminal stage with haematogenic metastases. In 6 patients suspected at first of having a tumour, the diagnoses could not be confirmed. The diagnosis in these cases were: volvolus (1), chronic gastritis with strictures (2), pyloric ulcer (1), renal cyst (1) and mastopathia cystica fibrosa (1). In addition, 6 healthy blood donors were examined.

Human encephalitogenic protein (HEP) was prepared from human brain (Caspary and Field, 1965). Carcinoembryonic antigen (CEA) was obtained from liver metastases of a colonic adenocarcinoma (Grossmann *et al.*, 1975).

Extracts in 3M KCl from surgically removed carcinomas (TAA) and from normal tissues (NTA) were prepared according to the method of Meltzer *et al.* (1971) with slight modifications described by Müller *et al.* (1975). They were taken from single carcinomas, except for bronchogenic carcinoma extract, which was pooled from an oat-cell carcinoma, an adenocarcinoma and a squamous-cell carcinoma. Extracts were lyophilized and dissolved immediately before use.

Leucocytes were obtained from buffy coat of heparinized blood by sedimentation with dextran. They were washed $\times 3$ and resuspended in Eagle's minimal essential medium (Institut für Nährmedizin und Immunpräparate, Berlin GDR) containing 10% human serum (pooled from AB donors) 66 u/ml penicillin and 66 $\mu$g/ml streptomycin.

Cell suspension in 40 $\mu$l ($2{\cdot}5 \times 10^5$ leucocytes/$\mu$l) were added to separate

Correspondence to: Dr med. Frank Emmrich, Bereich Medizin der Karl-Marx-Universität, Medizinische Klinik, Immunologische Forschungsgruppe, DDR-701 Leipzig, Johannisalle 32.

Correspondence on MEM test to: Prof Dr sc. med. Martin Müller, Medizinische Akademie "Carl Gustav Carus" Dresden, Pathologisches Institut, DDR-8019 Dresden, Fetscherstrasse 74.

test tubes ("U" microtitre plates may be used), mixed with 10 $\mu$l of the antigen solution or a PBS control, and incubate for 1 h at 37°C. Antigen protein was used at the rate of 100 $\mu$g per leucocyte sample.

Fresh agarose plates (0·9%) were prepared by mixing a doubly concentrated agarose solution at 48°C with the same quantity of medium with 10% human serum, and pouring the mixture on to glass plates placed in rectangular trays. Thirty holes were cut in each agarose plate with a 2·5mm hypodermic needle, using a stencil. After that, the contents of each tube were resuspended and 10 $\mu$l were put into the wells of agarose plates in triplicate with a triplicate control on each plate. Plates were then placed in moist chambers for 18 h at 37°C. Agarose plates were fixed with methanol for 30 min followed by 35% formalin for 30 min. Then the agarose layer was gently removed, glass plates were air-dried and migration areas were analysed by means of a (9 × 12) cm slide projector. The migration index (MI) was calculated as the ratio between average areas of cultures containing antigen (TAA) and control cultures on each plate. Results were analysed by Student's *t* test to establish *P* values. In preliminary studies, the mean value ± s.d. of the MIs in 6 healthy blood donors and in 6 non-tumour patients was 0·83–1·20 at 100 $\mu$g TAA. MI values of 0·80 or less and above 1·20 were considered significant. Values from 0·79 to 0·80 were taken as borderline.

The method for the MEM test has been described in detail elsewhere (Irmscher *et al.*, 1975; Müller *et al.*, 1975). In brief, human lymphocytes were prepared from defibrinated blood (Hughes and Caspary, 1970). After washing, the lymphocytes were resuspended in Eagle's Minimal Essential Medium distributed to test tubes ($10^6$ lymphocytes/2·5 ml) and incubated with 100 $\mu$g protein of several KCl extracts (0·1 ml) for at least 90 min at 37°C. Then supernatants were removed and mixed with irradiated macrophage suspension ($10^7$ cells in 0·5 ml). The electrophoretic mobility of 25–40 cells per sample was measured simultaneously in 2 cytopherometers (Opton, Oberkochem, FRG) with a maximum of tolerated time difference of 20%. The times measured for single cells of one sample were averaged and percentage of slowing was calculated as

$$\frac{\text{mean time of test mixture} \times 100}{\text{mean time of all samples without slowing}}$$

The results were calculated with a computerized *t*-test programme, and slowings were considered to be significant if *P* was $< 0·05$. On an experimental basis the slowings were interpreted as follows: $<5\%$ = no effect; 5–10% = weak result, estimated ultimately by calculation of significance, $>10\%$ = strong positive result.

Blood samples from 21 patients and 6 healthy blood donors were investigated with the same antigens simultaneously in LMI and MEM tests (Figs. 1–4). In 15/21 cases, malignant tumours were confirmed by histological examination. Fig. 1 shows the 15 cases tested with tumour extracts of the same individual tumour type. Ten cases could be found to agree in positive reactions. Three cases of renal carcinoma reacted only in the MEM test with the kidney carcinoma extract, but in the LMI, 2 of these reacted with the corresponding normal tissue antigens (NTA). One case of gastric carcinoma only reacted in the LMI with the gastric carcinoma antigen which was common to both test systems. Another gastric carcinoma extract, used only in the MEM test, and a colonic carcinoma extract, gave full MEM reaction in this case. One case of colonic carcinoma failed to react with colon cancer extract in both systems. KCl extracts expected to be negative are compared in Figs. 2 and 3, but only cases showing lack of correspondence were marked. Only 2 cross-reactions with $>10\%$ macrophage slowing could be seen in the MEM test (Fig. 2, 3) both in cases of gastrointestinal cancer. In LMI the number of cross-reactions with NTA and non-corresponding TAA was greater than in

MEM, but 7/9 cross-reacting NTA extracts had the same tissue type as the tumour. CEA was tested in both gastrointestinal and non-gastrointestinal carcinomas (Fig. 4). Five out of 6 gastrointestinal carcinomas were recognized by the MEM test and 4/6 by LMI. One case of a gastric carcinoma arising from a gastric ulcer

FIGS. 1–3.—LMI compared to MEM. Reactivity against TAA and NTA in 15 cases of carcinoma tested in LMI and MEM tests. Abscissa indicates the MI difference from 1·00 (× 100) recording stimulation as well as inhibition; the ordinate indicates the percentage of macrophage mobility inhibition.
● ○, colonic; ▲ △, gastric; ■ □, renal; ▼ ▽, bronchogenic; (Black TAA, White NTA) ×, mammary TAA. Hatched regions include all other antigens tested.

Fig. 1.—Results with allogeneic corresponding TAA (TAA of patients' tumour type).

Fig. 2.—Results with allogeneic TAA of another tumour type. (Cross-reactivity).

Fig. 3.—Results with allogeneic NTA (in LMI all except two NTA had the same tissue type as the tumour in that case). (Cross-reactivity).

gave negative results in both tests. One patient with a kidney carcinoma showed an MI value with CEA which could not be correlated with the MEM test. A further positive LMI with CEA was caused by a carcinoma of the gallbladder. Common examinations with HEP in 6 tumour patients showed significant reduction of macrophage mobility in the MEM test, but neither in these cases nor in additional cases examined only in LMI could migration inhibition be observed. Six patients suspected of tumour before biopsy and 6 healthy blood donors showed no migration effect with any antigen tested, with the exception of one borderline

FIG. 4.—Reactivity against CEA in 15 cases of carcinoma tested for LMI and MEM (×). ⊗ gastric carcinoma; ⊗ colonic carcinoma.

reaction in a case of clinically suspected mammary carcinoma, which showed an MI of 0·80 with kidney carcinoma extract.

In comparison with the Clausen assay, the MEM test consumes more time and needs specialized equipment and experienced investigators, but could be shown to be a highly specific test system (Irmscher *et al.*, 1975; Müller *et al.*, 1975; Seyfarth *et al.*, 1976). The Clausen assay is very suitable for clinical studies detecting antitumour reactivity, because of the simple cell-separation procedure, the inexpensive fitting-out and the quick evaluation and preserving method. A further advantage could be provided by using large culture plates, thus giving the same prerequisites to a great number of different test samples. Bergstrand *et al.* (1974) performed an analysis on the methodological variability of LMI under agarose. They showed that technical errors in the method can be reduced to a reasonable level, making it possible to observe relatively small specific effects of antigen.

In our study, 14/15 carcinomas could be detected and localized using the corresponding cancer extract, and partly by reactions with allogeneic NTA.

By use of a battery of antigenic preparations, some tumours which fail to be detected by a single antigen can be indicated in a comparison of different TAA and NTA preparations in different test systems. Previously, HEP was reported to be an antigen which caused reaction in a great number of tumour patients (Irmscher *et al.*, 1975; Meyer-Rienecker *et al.*, 1974; Müller *et al.*, 1975). We could not confirm this finding in LMI performed simultaneously with positive MEM test. Bergstrand *et al.* (1974) also described negative results with HEP in 7/9 carcinomas (LMI).

Using KCl extracts for antigens, the MEM test seems to be superior to the LMI in respect of its specificity, but LMI can be helpful in laboratories not possessing the equipment necessary for the MEM technique. Because of the considerable number of false-negative results previously reported, a great number of repeatedly proved and well-defined antigens in few standard concentration steps must be used to avoid false-negative results. Then LMI shows sufficient sensitivity, but one needs a very simple technique, for instance Clausen's, to perform the great number of single tests needed in each "checking programme".

The authors thank Dr Gold, Montreal, for testing our CEA substances.

We wish to thank also Institut für Impfstoffe, Dessau, GDR, for help and support in preparing the KCl extracts.

### REFERENCES

Bergstrand, H., Källén, B. & Nilsson, O. (1974) Effect of Basic Encephalitogenic Protein and some Peptides Derived from it on the Migration in Agarose Gel of Leukocytes from Patients with Multiple Sclerosis, other Neurological Diseases, or Carcinoma. *Acta neurol. scand.*, **50**, 227.

Boddie, A. W., Jr., Holmes, E. C., Roth, I. A. & Morton, D. L. (1975) Inhibition of Human Leukocyte Migration in Agarose by KCl Extracts of Carcinoma of the Lung. *Int. J. Cancer*, **15**, 823.

Caspary, E. A. & Field, E. J. (1965) An Encephalitogenic Protein of Human Origin; some Chemical and Biological Properties. *Ann. N.Y. Acad. Sci.*, **122**, 182.

Caspary, E. A. & Field, E. J. (1970) Sensitization of Blood Lymphocytes to Possible Antigens in Neurological Diseases. *Eur. Neurol.*, **4**, 257.

Clausen, J. E. (1971) Tuberculin-induced Migration Inhibition of Human Peripheral Leucocytes in Agarose Medium. *Acta allergol.*, **26**, 56.

Cochran, A. J., Mackie, R. M., Thomas, C. E., Grant, R. M., Cameron-Mowat, D. E. & Spilg, W. G. S. (1973) Cellular Immunity to Breast Carcinoma and Malignant Melanoma. *Br. J. Cancer*, **28**, Suppl. 1, 77.

Grossmann, H., Heidl, G. & Müller, M., (1975) Zur Isolierung des Carcinoembryonalen Antigens. *Acta biol. med. germ.*, **34**, 1347.

Hughes, D. & Caspary, E. A. (1970) Lymphocyte Transformation *In vitro* Measured by Tritiated Thymidine Uptake. *Int. Arch. Allergy Appl. Immunol.*, **37**, 506.

Irmscher, J., Müller, M., Fischer, R., Otto, G. & Strietzel, M. (1975) Makrophagen-Elektrophorese-Mobilitäts-Test (MEM) zur Immunologischen Diagnose Maligner Geschwülste. *Dt. Gesundh.-Wesen*, **30**, 687.

Kjaer, M. (1975) The Dose-related Effect of Tumour Extract on the *In vitro* Migration of Leukocytes from Patients with Renal Carcinoma. *Eur. J. Cancer*, **11**, 281.

McCoy, J. L., Jerome, L. F., Dean, J. H., Perlin, E., Oldham, R. K., Char, D. H., Cohen, M. H., Felix, E. L. & Herberman, R. B. (1975) Inhibition of Leukocyte Migration by Tumor-associated Antigens in Soluble Extracts of Human Malignant Melanoma. *J. natn. Cancer Inst.*, **55**, 19.

MELTZER, M. S., LEONHARD, H. J., RAPP, H. J. & BORSOS, T. (1971) Tumor-specific Antigens Solubilized by Hypertonic Potassium Chloride. *J. natn. Cancer Inst.*, **47**, 703.

MEYER-RIENECKER, H., JENSSEN, H. L., KÖHLER, H. & GÜNTHER, J. K. (1974) Makrophagen-Elektrophorese-Mobilitäts-Test (MEM) bei Malignen Neoplasmen des Zentralnervensystems. *Dt. Gesundh.-Wesen*, **29**, 844.

MÜLLER, M., IRMSCHER, J. FISCHER, R. & GROSSMANN, H. (1975) Immunologisches Tumorprofil. Ein Neuartiges Prinzip in der Anwendung des Makrophagen-Elektrophorese-Mobilitätstest (MEM) zur Differenzierten Karzinomdiagnose. *Dt. Gesundh.-Wesen*, **30**, 1836.

SEYFARTH, M., JENSSEN, H. L., WERNER, H., KÖHLER, H., JASTER, D., SEYFARTH, H. & HÖHNDORF, H. (1976) Immundiagnostik bei Knochentumoren. *Dt. Gesundh.-Wesen*, **31**, 1258.

TAUTZ, C., KHOEN, F. & AX, W. (1974) Human Leucocyte Migration under Agarose: Migration Inhibition by Soluble and Tumor Antigens with Special Reference to Antimelanoma Activity in Healthy Blacks. *Z. Immun.-Forsch.*, **147**, 155.